# Remembering Professor Benito Casu (1927–2016)

**DOI:** 10.3390/molecules23020292

**Published:** 2018-01-31

**Authors:** Giangiacomo Torri, Giuseppe Cassinelli

**Affiliations:** Carbohydrate Sciences Department of Istituto di Ricerche Chimiche e Biochimiche “G. Ronzoni”, 20133 Milan, Italy; ronzoni@ronzoni.it

Heparin and related drugs have contributed in so many different ways to the drug discovery process, and have provided a platform to understand the pathophysiology of vascular and inflammatory diseases for nearly 100 years. Despite its discovery in 1917 by Jay McLean, then a medical student, the scientific and clinical progress in the understanding of heparin and related drugs has continued to expand. This Special Issue of *Molecules* was developed in commemoration of the 100-year Anniversary of the discovery of Heparin. It would have been appropriate to have the lead article in this issue be by Professor Benito Casu, one of the lead pioneers who laid the foundation for the understanding of the heparin structure and function. Unfortunately, professor Casu unexpectedly and regrettably passed away on 11 November 2016. His legacy as a teacher, scientist, leader and a visionary who led a group of scientists at Ronzoni to advance the science of glycosaminoglycans will live for years to come. His diverse interest in this area is well represented in the manuscripts in this Special Issue.

At the G. Ronzoni Institute in Milan (Italy), he contributed over the last sixty years to the advancement of the knowledge of polysaccharide chemistry and biochemistry, especially both heparin and glycosaminoglycan (GAG) derivatives, analogs and mimetics. Several of these resulted from translational networks fruitfully set up by Professor Casu, who had the merit of sharing intuitions and projects with scientists, academic institutions, and industry. He had a unique attitude towards “*friendly competition*”, regardless of potential competitors, and used to cite a phrase of the Nobel Prize winner Rita Levi Montalcini: “*Research is a tool of knowledge and not a matter of power and competition*”.

In 1951, he started his scientific career with brilliant research on starch and cyclodextrins at the G. Ronzoni Institute. In 1969, he was first introduced to heparin during a sabbatical year at McGill University, Montreal (Canada), joining the group of Professor Arthur Perlin. The pioneer NMR studies on the structure and conformational flexibility of heparin provided him an international notoriety [1,2,3]. For over forty years, under his research coordination and operative direction, the Institute, through interdisciplinary and international networks and collaborations, significantly contributed to the development of both new analytical methodologies and novel heparin derivatives. This area of research provided a unique platform for research and education. This was the result of national and international exchange of students and senior scientists, all having the opportunity of sharing time and talent to advance the glycosciences. He promoted and organized, with his colleague Job Harenberg (Heidelberg-Mannheim University), the “1st Symposium on Glycosaminoglycans” at the Villa Vigoni, Loveno, Lake of Como (Italy) in 1991. The following twenty-four yearly symposia were always the preferred platform for pioneers, scholars and young investigators to present the more advanced research and multidisciplinary studies in the field. The 25th-Anniversary Symposium, in September 2017, was dedicated to Professor Casu.

In the framework of this preface, it is not possible to assess the content and richness of ideas, publications, reviews and patents of Prof. Casu. As a result of translational collaborations with academic institutions and/or industrial partners, the following highlights can be underscored:

- Structural characterization studies of heparin pentasaccharide sequence binding to antithrombin, fundamental for anticoagulant activity [4].
- Bioactive biotechnological heparins obtained by chemo-enzymatic modification of a biosynthetic precursor of bacterial origin [5].
- An honorary doctorate of the Uppsala University (Sweden), awarded in 1998 for outstanding contribution to heparin and GAGs knowledge and development.
- More recently, as a result of an international collaboration among G. Ronzoni Institute, Alabama University, USA (Prof. Sanderson), and Technion University, Haifa (Israel), granted by NCI, USA, the identification of a new class of non-anticoagulant heparins endowed with antineoplastic activity through the inhibition of heparanase [6,7], currently a lead compound under ongoing clinical trials.

The traditional interdisciplinary and international network of the Ronzoni Institute, under the guidance of Professor Casu and his group, is well expressed by the contributions of this special issue covering some of the translational steps “from bench to bedside”.
